# iMSDE improves the fat suppression efficiency in vessel wall imaging

**DOI:** 10.1186/1532-429X-13-S1-P364

**Published:** 2011-02-02

**Authors:** Jinnan Wang, Vasily L Yarnykh, Peter Boernert, Chun Yuan

**Affiliations:** 1 Philips Research North America, Seattle, WA, USA; 2 University of Washington, Seattle, WA, USA; 3 Philips Research Europe, Hamburg, Germany

## Objective

To explore the suppression efficiency of spectrally selective fat suppression schemes when different black blood imaging pre-pulses are used.

## Background

Sufficient peri-vascular fat suppression is critical for the outer wall boundary delineation in vessel wall imaging. The MSDE technique has been shown to significantly improve the blood suppression efficiency and measurement reproducibility. A limitation is that, due to the uncompensated eddy currents, undesired frequency shift can be observed after the MSDE prepulse, making the spectrally selective fat suppression ineffective. The aim of this study is to explore whether a previously proposed improved MSDE (iMSDE) technique is more robust against this system imperfection than the conventional MSDE approach, thus improving the fat suppression efficiency in vivo.

## Methods

A computer simulation program was used to examine the frequency shift induced by the different pre-pulses. Parameters from a number of clinical carotid scan settings (first gradient moment m_1_ ranges 185-2280 mTms^2^/m) were used for the simulation. Three healthy volunteers and one patient with diagnosed carotid artery atherosclerosis disease were recruited for carotid artery scans using both techniques. The imaging parameters used for both scans were identical except the pre-pulse itself. Specifically, TSE, TR/TE 4800/10ms, FOV 160×160mm^2^, in-plane resolution: 0.6×0.6mm^2^, slice thickness: 2mm, ETL: 12, fat suppression. ROIs were carefully delineated on peri-vascular fat and air on matched MR images. The SNR of the peri-vascular fat was measured for each subject.

## Results

**Figure 1:**
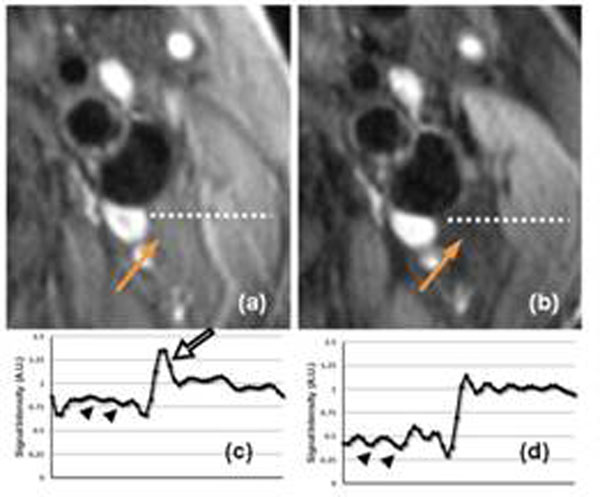
In vivo images acquired on an atherosclerosis patient using both MSDE (a) and iMSDE (b) techniques. Notice the dramatic signal difference on the fat region on both images (arrows). The signal intensity profiles along the dotted lines were plotted (c.d) for both images. Adter normalizing to the muscle signal, significantly higher fat signal was found in the MSDE case, compared to the iMSDE (arrow heads). Also notice the signal peak caused by the chemical shift from unsuppressed fat (open arrow).

In a carotid artery scan, the MSDE prepulse causes 1.7-6.3 PPM frequency shift, while the iMSDE causes only 0.1-0.7PPM. Considering the water-fat separation of 3.5PPM, the MSDE prepulse causes a significant fat frequency shift that causes insufficient fat saturation, while the iMSDE doesn’t. For in vivo comparisons dramatically improved saturation was observed in all iMSDE images when compared with the corresponding MSDE images (Fig.1 (a, b)). Quantitatively, the mean fat SNR is dramatically lower on the iMSDE images (8.6±2.6) than on the MSDE images (13.2±2.4). This can also be observed when the signal intensity profile is plotted and normalized to muscle (Fig.1 (c, d) ).

## Conclusion

In this study, the spectrally selective fat suppression technique’s performance on MSDE and iMSDE was studied. Both the computer simulation and in vivo measurements suggested that the iMSDE technique provides a more robust fat suppression for vessel wall imaging. This improvement shows that the iMSDE technique can provide more accurate and potentially more reproducible outer wall boundary measurements in vessel wall imaging.

